# Using risk adjustment to improve the interpretation of global inpatient pediatric antibiotic prescribing

**DOI:** 10.1371/journal.pone.0199878

**Published:** 2018-07-06

**Authors:** Julia A. Bielicki, Mike Sharland, Ann Versporten, Herman Goossens, David A. Cromwell

**Affiliations:** 1 Paediatric Infectious Diseases Research Group, Infection and Immunity, St George’s University of London, London, United Kingdom; 2 Department of Health Services Research and Policy, London School of Hygiene and Tropical Medicine, London, United Kingdom; 3 Paediatric Pharmacology Group, University of Basel Children’s Hospital, Basel, Switzerland; 4 Laboratory of Medical Microbiology, Vaccine & Infectious Disease Institute (VAXINFECTIO), Faculty of Medicine and Health Science, University of Antwerp, Antwerp, Belgium; National Yang-Ming University, TAIWAN

## Abstract

**Objectives:**

Assessment of regional pediatric last-resort antibiotic utilization patterns is hampered by potential confounding from population differences. We developed a risk-adjustment model from readily available, internationally used survey data and a simple patient classification to aid such comparisons.

**Design:**

We investigated the association between pediatric conserve antibiotic (pCA) exposure and patient / treatment characteristics derived from global point prevalence surveys of antibiotic prescribing, and developed a risk-adjustment model using multivariable logistic regression. The performance of a simple patient classification of groups with different expected pCA exposure levels was compared to the risk model.

**Setting:**

226 centers in 41 countries across 5 continents.

**Participants:**

Neonatal and pediatric inpatient antibiotic prescriptions for sepsis/bloodstream infection for 1281 patients.

**Results:**

Overall pCA exposure was high (35%), strongly associated with each variable (patient age, ward, underlying disease, community acquisition or nosocomial infection and empiric or targeted treatment), and all were included in the final risk-adjustment model. The model demonstrated good discrimination (c-statistic = 0.83) and calibration (p = 0.38). The simple classification model demonstrated similar discrimination and calibration to the risk model. The crude regional pCA exposure rates ranged from 10.3% (Africa) to 67.4% (Latin America). Risk adjustment substantially reduced the regional variation, the adjusted rates ranging from 17.1% (Africa) to 42.8% (Latin America).

**Conclusions:**

Greater comparability of pCA exposure rates can be achieved by using a few easily collected variables to produce risk-adjusted rates.

## Introduction

Antibiotics are among the most commonly used medications for hospitalized children [1]. On any day, 30% to 60% of children admitted to hospital around the world will receive at least one antibiotic, with many being prescribed multiple systemic antimicrobials [2,3].

Antimicrobial stewardship interventions can improve antibiotic use in this vulnerable population and are usually implemented at a high level of aggregation, for example at hospital level [4,5]. It is often desirable to compare the use of antibiotics, especially of last-resort agents, between hospitals or regions to identify outliers and therefore areas for intervention. However, merely comparing the overall volume of use or crude proportions for antibiotics of interest is unlikely to be useful because prescription patterns vary markedly, and this is partially due to differences in patient case-mix [6–11].

In many areas of infection control, regression models are used to adjust metrics for differences in patient case-mix [12–14]. However, these risk-adjustment models can easily become complex, may be based on specific data that are not widely available and/or comparable, and can require the adoption of extensive, costly data collection processes.

Another method is to apply a stratification system and examine exposure within groups of similar patients. An example of this method from another area of medical practice is the Robson classification, which stratifies pregnant women according to simple and widely available clinical characteristics that influence their a priori risk of having a Caesarean delivery [15–17].

We examined whether a risk-adjustment model could be developed from readily available variables that would facilitate the fair comparison of statistics from point prevalence surveys (PPS) on the prescribing of antibiotics to children with sepsis/bloodstream infections. We focused on three “pediatric conserve antibiotics” (pCAs) for severe Gram-negative and Gram-positive neonatal and pediatric infections. These antibiotics are part of the newly defined World Health Organization Watch group of antibiotics. This group has been identified to have a higher resistance potential, and should only be used for specific indications or in infections caused by bacteria suspected or proven to be resistant to less broad-spectrum options [18]. We evaluated whether available variables enabled the creation of: (i) a risk-adjustment model to fairly compare the prevalence rates across world regions, and (ii) a simple stratification system that identified patient groups who would be expected to have similar exposures due to their characteristics.

## Materials and methods

### Data collection

The study used data collected as part of the Antibiotic Resistance and Prescribing in European Children (ARPEC) project global PPS [3]. PPS are simple, standardized tools used widely internationally to collect data on antimicrobial use to facilitate monitoring within centers and countries [19]. Participating centers were asked to conduct a one-day cross-sectional survey of antimicrobial prescriptions for inpatients on neonatal and pediatric wards during three periods in 2011/2012 [2,3]. During each PPS all neonatal and pediatric wards in participating institutions had to be surveyed once within the defined auditing period. All patients present in the wards at 8:00 am, and at least since midnight on the day of the survey, were recorded. For each patient treated with at least one antimicrobial, detailed data on the prescription as well as about the patient were collected according to a standardized protocol.

The ARPEC PPS were conducted in 226 participating centers located in 41 countries, which were grouped into continental regions (Africa, Asia, Australia, Europe–East, Europe–North, Europe–South, Europe-West, Latin/South America and North America) according to the UN geoscheme classification [2,3].

The PPS methodology and data collection approaches have previously been described in detail [2,3]. During data collection no unique identifiers, such as hospital numbers or dates of birth, were recorded. As the PPS was therefore a completely anonymized audit of antimicrobial prescribing to inpatient neonates and children, formal ethical review was not a requirement. Individual participating centres were asked to ascertain any local requirements for ethical review. By entering data, centres confirmed that they had taken the required steps according to their local and national regulatory and legal requirements.

### Study population and definition of patient and treatment characteristics

The study used the records of surveyed patients who were prescribed systemic antibiotics (J01) [20] for the most common indication of suspected or definitive sepsis/bloodstream infection [3], excluding febrile neutropenia and catheter-related bloodstream infection. A single key infection syndrome was selected as different factors may drive prescribing of antibiotics depending on the type of infection being treated. Relevant prescriptions were identified from the PPS information on “reason for prescription”.

In terms of antibiotic use, we focused on carbapenems (J01DH), glycopeptides (J01XA) and linezolid (J01XX08). Prescribing of these antibiotics may reflect actual or feared infection caused by resistant organisms, such as extended-spectrum beta-lactamase producing Gram-negative bacteria or methicillin-resistant *Staphylococcus aureus*. The World Health Organization confirms these antibiotics, among others, as key targets for national antibiotic stewardship [18]. Our study is limited to the indicated groups and follows the same approach as a recent study evaluating the impact of antimicrobial stewardship on antibiotic prescribing in US children’s hospital [21]. Exposure to pCAs was defined at the patient level, with a patient classified as exposed if one or more of the antibiotics listed above was prescribed.

At the patient level, the ARPEC dataset included information on a patient’s age, whether they had any chronic conditions, and the type of ward the patient was on. Data were also collected on the type of prescription (empiric or targeted). Neither the microbiological results for individual patients nor hospital antibiograms were available. Finally, timing of prescription was available as having been issued >48 hours after hospitalization (hospital-acquired) or ≤48 hours after hospitalization (community-acquired). Any prescription for sepsis/bloodstream infection in the first three days of life was considered neonatal early onset sepsis. Wards were classified as either a neonatal intensive care unit (NICU, all care levels), pediatric intensive care unit (PICU) or other pediatric wards. Patients with any recorded underlying disease from a predefined list including surgical malformations, chronic neurological, gastrointestinal, endocrine, lung and renal disease as well as congenital heart disease, oncologic/hematologic diseases, genetic or metabolic disorders, rheumatological or autoimmune disease and chronic infections were labeled as having underlying disease (S1 File). Patients receiving any targeted prescriptions for a sepsis/bloodstream infection (according to the ARPEC protocol based on pathogen identification and/or antimicrobial susceptibility testing) were defined as receiving targeted treatment, even when additional prescriptions were empiric. All other patients were labeled as receiving empiric treatment.

### Statistical analysis

Logistic regression was used to assess the association between pCA exposure and the individual patient and treatment characteristics. Age was dichotomized into neonates aged 3 days or younger versus infants aged 4 days or older and children (reflecting clinical differences between early-onset and late-onset sepsis among neonates).

We then developed a risk model using multivariable logistic regression.

The model was developed by sequentially adding each available patient variable, starting with the variable that had the strongest univariate association and ending with the weakest. A Wald test was used to assess the contribution of an added variable to the model and a p value of 0.05 was used as the threshold for inclusion. Following this, interactions between included variables were explored. The performance of the model was assessed in terms of its calibration and discrimination. Calibration describes the level of agreement between the predicted and observed risks, and was evaluated using the Hosmer-Lemeshow test. Discrimination indicates the ability of a model to distinguish patients with a lower and higher risk of pCA prescription. We evaluated this by using the c-statistic (equivalent to the Area under the ROC curve).

The regression model was used to calculate risk-adjusted regional pCA exposure rates. These were derived using indirect standardization, which involved multiplying the ratio of observed/expected exposure rates by the mean exposure rate in the whole cohort [9]. Approximate 95% confidence intervals were derived for proportions and indirectly standardized rates using the Wilson Score and Byar’s Method, respectively.

As a sensitivity analysis, we repeated the above process using a multilevel logistic model, which incorporated a random-intercept term for the subregions as well as the explanatory variables. This accounted for any lack of independence in the data due to patients being clustered within subregions.

Finally, a small number of mutually exclusive and comprehensive patient subgroups were defined on the basis that they described clinical situations in which we would expect a patient’s chance of receiving pCA to be similar given the seriousness of the situation and the effectiveness of current antiobiotics. We used the same variables that were considered in the risk-model development process because they represented information that is easy to collect and can be standardized. Inspection of the variables identified six patient groups that were expected to be associated with different levels of exposure to pCA:

1. Neonatal early onset sepsis (infants ≤3 days of age): High reported coverage provided by narrow-spectrum regimens [22].
2. Community-acquired sepsis in otherwise healthy infants >3 days of age and children: Lower levels of colonization and infection by multidrug-resistant pathogens [23].
3. Community-acquired sepsis in infants >3 days of age and children with underlying disease: Colonization by multidrug-resistant pathogens possible with reported epidemiology similar to hospital-acquired bloodstream infection [23].
4. Empiric treatment of hospital-acquired sepsis in infants and children of any age outside of PICU: Colonization by multidrug resistant pathogens possible, but colonization pressure less than in intensive care [24].
5. Targeted treatment of hospital-acquired sepsis in infants and children of any age outside of PICU: May include patients having been discharged from intensive care to complete treatment after stabilization, therefore likely to partially reflect intensive care epidemiology [25].
6. Hospital-acquired sepsis in infants and children of any age in PICU: Colonization by multidrug-resistant bacteria expected with high colonization pressure in intensive care [25].

We examined the ability of these subgroups to reduce the heterogeneity within the patient population using the measures of discrimination and calibration described above. All statistical analyses were carried out using Stata/IC 13.1®, Statacorp, USA.

## Results

### Description of cohort

**Fig 1:**
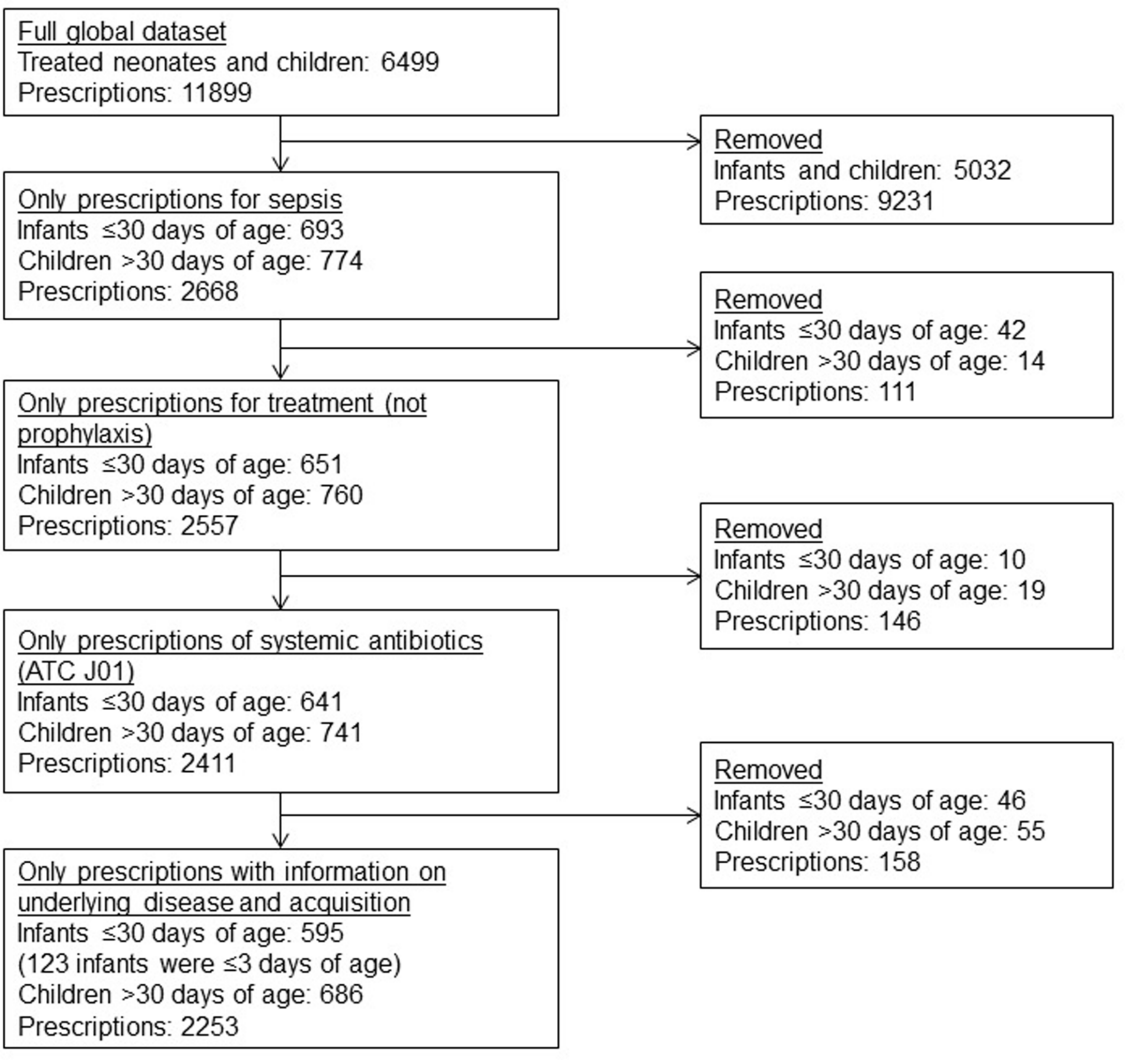
Flow chart of prescription and patient inclusion.

The complete global ARPEC PPS cohort contained data on 11899 prescriptions on 6499 patients. Among these, there were 2668 prescriptions for sepsis, but limiting the cohort to patients with complete records led to the exclusion of a further 415 prescription records (Fig 1). The final dataset contained 2253 systemic antibiotic prescriptions for 1281 infants and children, representing 19% of a total of 11899 recorded prescriptions.

### Overall pCA exposure

Of the 1281 included patients, 445 patients (34.7%; 159 children ≤30 days of age of which two were ≤3 days of age, 286 children >30 days of age) were exposed to pCAs. In total, 18.4% (235/1281) were receiving carbapenems, 25.4% (325/1281) glycopeptides and 1.2% (16/1281) linezolid. For each of the patient and treatment characteristics, the proportion of exposed patients varied across the levels of each variable by at least 10%, as shown in Table 1.

**Table 1:** Association of key patient characteristics with exposure to pCA antibiotics (group comparisons using Χ^2^ testing).

	Total patients with sepsis/BSI	% in group	pCA-exposed	% exposed	p-value
**Age**					
Neonate ≤3 days of age	123	9.6	2	1.6	p<0.001
Infant or child >3 days of age	1158	90.4	443	38.3	
**Ward**					
Pediatric ward	466	36.4	117	25.1	p<0.001
Neonatal intensive care	635	49.6	219	34.5	
Pediatric Intensive care	180	14.1	109	60.6	
**Underlying disease**					
Absent	311	24.3	32	10.3	p<0.001
Present	970	75.7	413	42.6	
**Acquisition of infection**					
Community	649	50.7	78	12.0	p<0.001
Hospital	632	49.3	367	58.0	
**Type of treatment**					
Empiric	980	76.5	285	29.1	p<0.001
Targeted	301	23.5	160	53.2	
**Total**	1281		445	34.7	

### Multivariable logistic regression model for exposure to pCA

Each individual patient and treatment characteristic was found to be associated with antibiotic use, and improved the performance of the multivariable logistic model when added (Table 1). There was evidence of an interaction between the variables “ward” and “acquisition of infection” as well as between the variables “underlying disease” and “type of treatment”. These separate variables were replaced by variables that captured the combination of categories. Table 2 shows the results of the model that takes into account these interactions. Overall, the following were associated with increased odds of pCA exposure: (1) presence of any underlying disease, (2) treatment in PICU; (3) receiving targeted treatment; (4) treatment for hospital-acquired infection. Being ≤3 days old was associated with lower odds of pCA exposure.

**Table 2:** Logistic regression results showing adjusted odds ratios for exposure to pediatric reserve antibiotics (pCAs) with 95% confidence intervals.

Group	Adjusted OR	95%CI
**Patients according to ward type and acquisition of infection**		
Non-ICU / community-acquired	Ref	-
Non-ICU / hospital-acquired	5.0	3.0–8.3
NICU / community-acquired	0.6	0.3–1.1
NICU / hospital-acquired	5.7	3.7–8.8
PICU / community-acquired	4.2	2.2–8.1
PICU / hospital-acquired	12.7	7.3–22.2
**Patients according to underlying disease and type of prescription**		
No underlying disease / empiric	Ref	-
No underlying disease / targeted	4.3	1.8–10.0
Underlying disease / empiric	3.8	2.2–6.7
Underlying disease / targeted	7.1	3.9–13.0
**Patients according to age**		
Neonate ≤3 days of age	Ref	-
Neonate >3 days of age, infant or child	16.9	4.0–70.9

**Fig 2:**
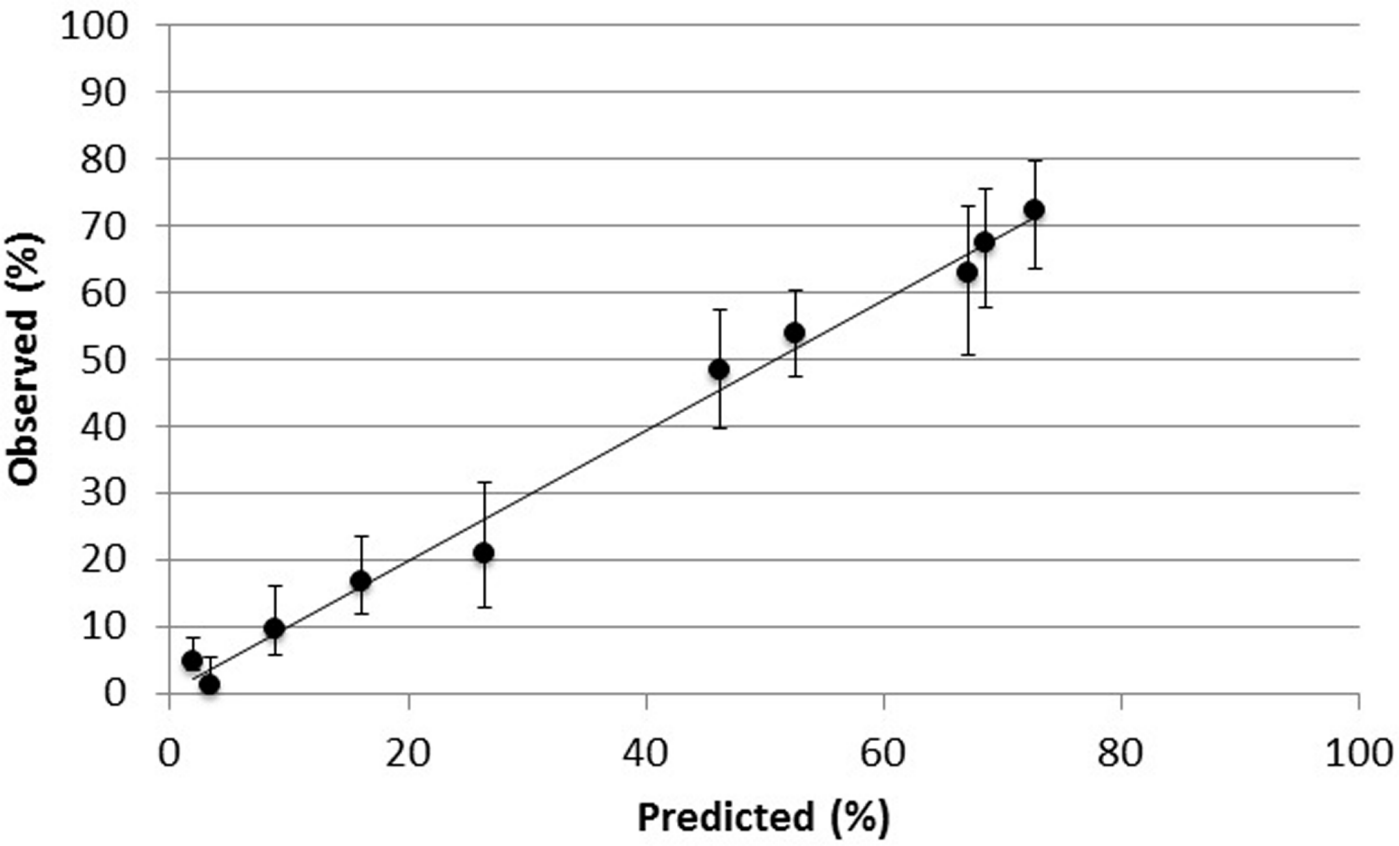
Calibration plot for logistic regression risk model of pCA exposure.

This final model demonstrated strong discrimination, with a c-statistic of 0.83. There was also evidence of good calibration (Hosmer-Lemeshow test, p = 0.38, see Fig 2 for calibration plot).

### Multilevel random-intercept logistic model for exposure to pCA

The analysis using the multilevel model gave similar results to the main analysis. We found only modest variation in the random-intercepts of the subregions (variance = 0.21; SE(var) = 0.14) and the coefficients of the explanatory variables were similar to those estimated in the standard model. In addition, the Pearson correlation coefficient between the predicted risks for individuals from the two models was 0.97, with the predictions from the multilevel model producing to almost identical calibration and discrimination figures.

### Adjusted regional patterns of pCA exposure

**Fig 3:**
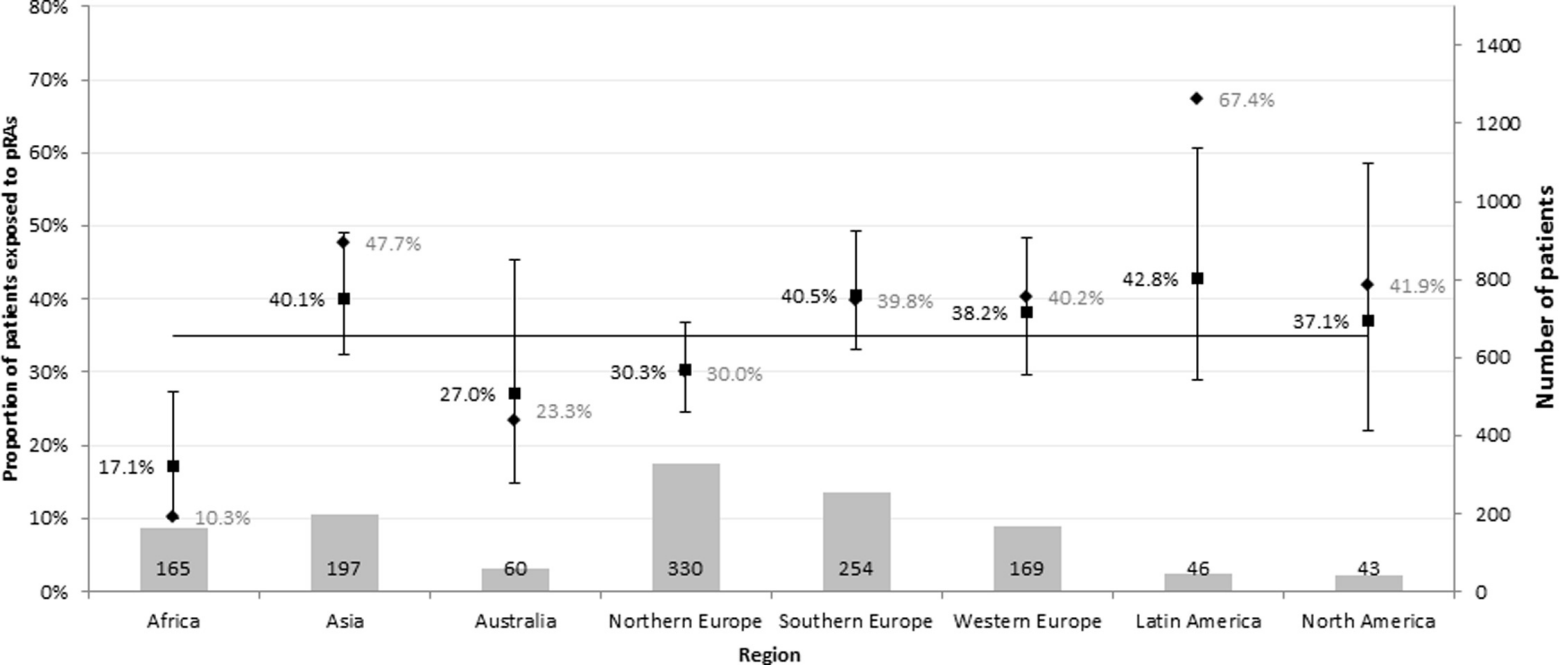
Crude and risk adjusted regional exposure rates for pediatric reserve antibiotics. Bars correspond to crude rates, squares to adjusted rates (shown with 95% confidence intervals). Data for Eastern Europe have been omitted due to low number of patients surveyed (n = 17) The horizontal line indicates the mean pCA exposure rate in the whole cohort. Patient numbers for each region are shown at the bottom of each bar.

Fig 3 demonstrates the impact of using the risk model to adjust for differences in patient characteristics on regional pCA exposure levels. Crude regional exposure rates ranged from 10.3% (Africa) to 67.4% (Latin America). After adjustment, there was substantially less variation between the regions, with the adjusted regional exposure rates ranging from 17.1% (Africa) to 42.8% (Latin America). The 95% confidence intervals around adjusted pCA exposure rates indicate that, with the exception of Africa, regional estimates may not differ from the overall cohort mean pCA exposure level once key characteristics have been taken into account.

### pCA exposure in predefined groups

Table 3 shows the characteristics of the six patient groups that were derived from clinical reasoning.

**Table 3:** Overall proportions of treated patients within predefined group and the expected rate of exposure to pediatric reserve antibiotics (pCAs).

	Patient group	Total patients (n)	% in group	Exposed to pCAs (n)	% Exposed	95% CI
**1**	Neonatal early onset sepsis	123	10%	2	1.6%	0.2 to 5.8
**2**	CA sepsis/BSI in otherwise healthy infants and children	251	20%	17	6.8%	4.0 to 10.6
**3**	CA sepsis/BSI in infants and children with underlying disease	295	23%	60	20.3%	15.9 to 25.3
**4**	Empiric treatment of HA sepsis/BSI outside of PICU	327	25%	162	49.5%	44.0 to 55.1
**5**	Targeted treatment of HA sepsis/BSI outside of PICU	173	13%	120	69.4%	61.9 to 76.1
**6**	HA sepsis/BSI on PICU	112	9%	84	75.0%	65.9 to 82.7
		1281		445	34.7%	

CA: community-acquired, HA: hospital-acquired, BSI: bloodstream infection, PICU: pediatric intensive care unit.

Table 4 shows the distribution of patient groups by region.

**Table 4:** Distribution of included patients for 6 predefined groups by region.

	Patient group	Subregion															
		Africa		Asia		Australia		Northern Europe		Southern Europe		Western Europe		Latin America		North America	
		n	%	n	%	n	%	n	%	n	%	n	%	n	%	n	%
**1**	Neonatal early onset sepsis	17	10.3	12	6.1	10	16.7	43	13.0	18	7.1	18	10.7	0	0	5	11.6
**2**	CA sepsis/BSI in otherwise healthy infants and children	38	23.0	24	12.2	14	23.3	64	19.4	70	27.6	28	16.6	1	2.2	4	9.3
**3**	CA sepsis/BSI in infants and children with underlying disease	74	44.9	54	27.4	12	20.0	55	16.7	40	15.8	44	26.0	9	19.6	10	23.3
**4**	Empiric treatment of HA sepsis/BSI outside of PICU	24	14.6	54	27.4	15	25.0	102	30.9	65	25.6	30	17.8	14	30.4	16	37.2
**5**	Targeted treatment of HA sepsis/BSI outside of PICU	11	6.7	27	13.7	4	6.7	47	14.2	35	13.8	30	17.8	12	26.1	5	11.6
**6**	HA sepsis/BSI on PICU	1	0.6	26	13.2	5	8.3	19	5.8	26	10.2	19	11.2	10	21.7	3	7.0
Carbapenem exposure		17	10.3	57	28.9	5	8.3	44	13.3	53	20.9	33	19.5	20	43.5	3	7.0
Glycopeptide exposure		12	7.3	59	30.0	12	20.0	74	22.4	75	29.5	56	33.1	19	41.3	17	39.5
Total n		165		197		60		330		254		169		46		43	

CA: community-acquired, HA: hospital-acquired, BSI: bloodstream infection, PICU: pediatric intensive care unit. The proportions refer to contributions of each group for the region in question.

**Fig 4:**
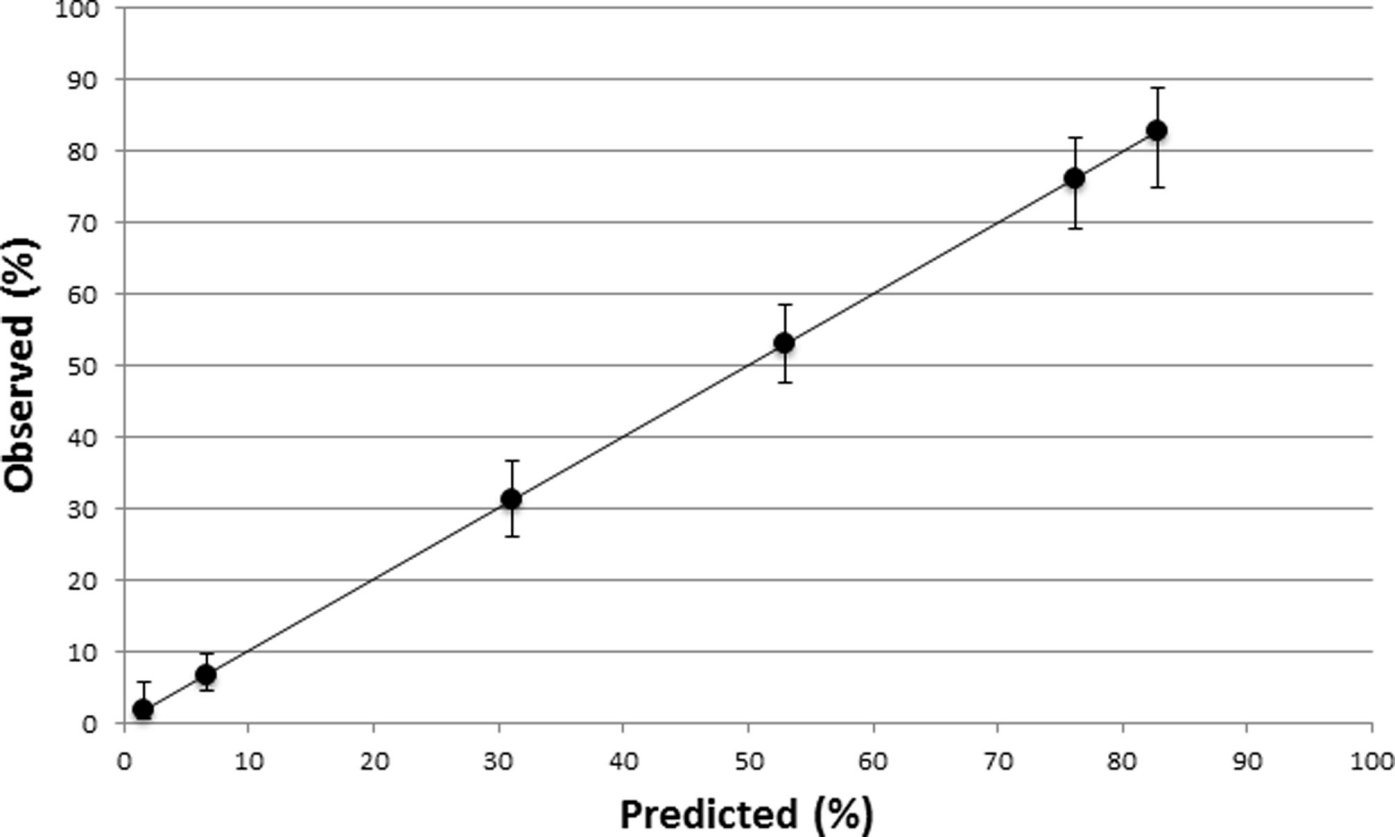
Calibration plot for logistic regression classification model of pCA exposure.

Overall, nearly 50% of children fell into groups 4 to 6, as they were being treated for hospital-acquired sepsis/bloodstream infection. In terms of the pCA exposure rates, levels were lowest in neonates treated for early onset sepsis (1.6%) and highest in patients with hospital-acquired sepsis on PICU (75.0%). We assessed the performance of this simple classification by using a logistic regression model that included only these six predefined groups. The model had a similar level of performance as the full risk model, with good levels of discrimination (c-statistic = 0.81) and calibration (Hosmer-Lemeshow test, p = 0.813; Fig 4). The regional distribution of patients may explain very high crude pCA exposure levels in Latin America: Nearly 80% of patients in this region fell into groups 4 to 6 compared with maximally 50–60% in other regions. These patients would be expected to have higher pCA exposure rates than patients in groups 1 to 3.

## Discussion

The data from global point prevalence surveys of inpatient neonatal and pediatric systemic antibiotic prescriptions for sepsis/bloodstream infection revealed large differences in the crude pCA prevalence rates across the regions. But, the interpretation of these differences is hampered by the considerable systematic differences between the regions in the patterns of disease, antimicrobial resistance and population structure. In this study, we demonstrated that having data on a few easily collected variables related to patient and treatment characteristics, it is possible to develop a risk adjustment model to produce adjusted pCA exposure rates, thereby allowing a fairer comparison of regions. In addition, the variables could be combined into a simple patient classification that differentiated various clinical situations in which the expected pCA exposure rates would be expected to differ. While a risk-adjustment approach based on logistic regression is preferable for making comparisons against a standard population, the classification facilitates benchmarking by creating relatively homogenous groups of patients who would be expected to have similar exposure to pCA due to their clinical circumstances. When evaluated, both approaches performed well at discriminating between children in terms of their likely exposure to pCA.

Overall, the average pCA exposure rate was high at 35%. Exposure rates to pCA were higher among older children, those on PICU, children with underlying disease, and receiving targeted treatment for hospital-acquired sepsis/bloodstream infection. That targeted treatment was strongly associated with higher pCA exposure may reflect a high rate of resistant bacteria identified in those children with culture-confirmed sepsis/bloodstream infection. Given the reported high rates of antimicrobial resistance in key pathogens globally, but especially in low- and middle-income countries [26–28], this is a worrying sign of the prevalence of multidrug-resistant infections, especially among hospital-acquired infections, in this population.

Regional crude prevalence rates varied considerably, the lowest and highest differing by a factor of 6.5. After adjustment, the prevalence rates varied by a factor of 2.5, demonstrating that a large proportion of variation arose from differences in the distribution of the measured patient and prescription characteristics. Previous analyses of case-mix adjustment in benchmarking of inpatient antibiotic prescribing have used variables that require detailed knowledge about each patient [7,9] or detailed hospital-level data [8,10,11]. While models based on these variables may have demonstrated even better discrimination and calibration in this dataset, the value of our study is to demonstrate that pCA prescribing rates from prevalence surveys can be adequately risk-adjusted using easily collected variables. The effectiveness of this approach needs to be replicated in other datasets, and the benefit of including other factors also needs evaluation. Nonetheless, the results underline the importance of focusing on the complete and accurate measurement of important patient-level variables and treatment characteristics during data collection to enable optimal utilization of PPS data.

The predefined patient groups based on clinical reasoning proved to have a similar level of performance to the full logistic regression model. In practice, the application of a logistic regression model to inform quality improvement at a hospital level could be challenging because it produces a single composite statistic that describes overall performance. In contrast, a classification-based approach makes it possible to monitor the prevalence of pCA in distinct types of patients, for which the action required to tackle above average rates is likely to be different. This has been found to be a key issue in the development and use of classification systems in other circumstances [15,16]. The clinical logic underpinning the classification gives it a face-validity that suggests it could be applicable in other situations. But, we recommend that, before it is adopted for use in other infection syndromes and healthcare settings (e.g. adult care), its performance is evaluated further using data collected in that setting.

Our analysis has a number of limitations. First, despite this being as far as we are aware the largest neonatal and pediatric antibiotic prescribing PPS database globally, some regions contributed only a small number of patients. With a larger sample size, we would have been able to better estimate true differences in regional pCA exposure rates. Sample size limitations will also impact the application of our approach at hospital-level. Assuming that prescribing practices remain relatively stable, the pooling of data from several PPS may be one approach to overcome small sample sizes. Second, we only included prescriptions that were recorded as being for sepsis/bloodstream infection. Patients in our cohort may have received additional antibiotics for another indication (e.g. lower respiratory tract infection), which we did not include in our evaluation. Whether the same risk factors are associated with pCA exposure in patients treated for other infections needs to be tested. Third, data on the causative organism in targeted treatment were not recorded. We therefore rely on local contributors having correctly identified the recorded treatment as the most suitable narrow-spectrum antibiotic option for the target pathogen. In the future, pCA exposure rates should be interpreted together with information on actual resistance at patient or aggregate levels [29,30] to gauge whether pCA exposure levels are high in response to high antibiotic resistance rates or are mainly driven by prescriber behavior. Fourth, PPS data provides no information on duration of pCA exposure, which may have an important impact on the volume of pCAs used in a specific setting. Fifth, our analysis would need to be repeated analyzing data from a variety of hospitals. ARPEC PPS participant centers were predominantly tertiary and/or university hospitals, and the relevance of our findings for benchmarking involving smaller secondary hospitals would have to be confirmed. Finally, the cluster sizes of the participating centers were too small to support a multilevel model with centre as the cluster. Instead, we fitted a random-intercept logistic model with subregions to take account into account the hierarchical structure of the data. This did not change the conclusions about each variable and there was excellent agreement between the predictions from the two models. We therefore chose to present the results from the simpler standard logistic model.

In addition to conventional case-mix adjustment approaches, predefined patient groups, such as those described in our analysis, enable the generation of aspirational targets for aggregate pCA exposure rates, either in local, regional or national settings. These targets could be based on current average levels of exposures or be based on expert consensus about desirable practice. This would allow the comparison of (i) overall standardized exposure rates; (ii) variations in distribution of patient strata; (iii) variations in exposure rates for specific patient groups. The advantages of this approach is that evaluations of pCA exposure would take into account key characteristics of the patients and infection episodes that are highly likely to influence pCA prescribing decisions and as such reflect justified use of these antibiotics. This may enable identification of specific target areas for intervention, while taking into account that what is appropriate may differ between facilities and/or regions. For such comparisons and target setting treatment and patient characteristics need to be captured, as described in this manuscript and as is standard during point prevalence surveys. Given the good performance of the logistic regression classification model in our analysis, the level of detail for the variables included in the ARPEC PPS may be sufficient for evaluations of childhood antibiotic use. However, additional or different variables are likely to be useful for similar analyses in other patient groups.

Case-mix adjustment, preferably using a few easy-to-collect patient and prescription characteristics, is key to accurately and fairly comparing prescribing patterns between health care providers, regional health care administrations and countries. Furthermore, assessing antibiotic exposure rates in a clinically relevant manner within homogenous and easily identifiable patient groups can be a rich source of information about key areas for intervention to improve antibiotic prescribing. Quality of antibiotic prescribing could then be assessed in such patient groups using validated indicators. In this way, interventions that will achieve a safe and reasonable reduction in the use of critically important antibiotics at aggregate level can be defined and evaluated.

## Supporting information

S1 File Excerpt of data collection instructions for coding of underlying disease for ARPEC PPS. (PDF)
