# Supplementary material for: Using risk adjustment to improve the interpretation of global inpatient pediatric antibiotic prescribing
Source: PLoS One. 2018 Jul 6;13(7):e0199878. doi: 10.1371/journal.pone.0199878 (PMC6034826; doi:10.1371/journal.pone.0199878)
Supplement: S1 File — (PDF) [file pone.0199878.s001.pdf]

Supporting Information

Excerpt of data collection instructions for coding of underlying disease for ARPEC PPS [2, 3].

Underlying diagnoses groups (paediatric patients):

Surgical disease/Malformations including all problems requiring surgical intervention/follow up, e.g. gut malformations/atresia, Urinary malformations, Sacral agenesis, Central Nervous System malformations, skin anomalies treated surgically including abscesses, any device insertion including gastrostomies, urinary catheter or Ventricular-peritoneal shunt, etc.

Chronic Neurological and Psychiatric disorders including Cerebral Palsy, Global Developmental Delay (GDD), all seizure disorders (epilepsy, West syndrome, etc.), progressive neurological and neuromuscular syndromes.

Gastroenterological disease including inflammatory bowel disorders, Gastrooesophageal reflux requiring treatment, Celiac disease, chronic non-infectious liver diseases, etc.

Congenital Heart Disease (CHD) including all the cardiac malformations and acquired cardiac disease e.g. Kawasaki syndrome, and cardiac surgery

Oncologic/Hematologic diseases and Bone Marrow Transplantation except immune deficiencies unless after bone marrow transplantation and all Solid Organ Transplantation.

Chronic Endocrinological Diseases including Cushing syndrome, thyroid disorders, pituitary gland disorders, etc.

Chronic Renal Disease, including Vesico-ureteric reflux.

Chromosomal/Single gene/Metabolic disorders (diabetes).

Rheumatological, autoimmune and chronic inflammatory diseases such as LED, sarcoidosis etc.

Chronic lung diseases including cystic fibrosis and chronic lung disease in ex preterm patients.

Chronic infectious diseases such as HIV, tuberculosis with ongoing treatment and chronic hepatitis B or C infection or primary immunodeficiencies.

Underlying diagnoses groups (neonatal patients):

Maternal prolonged rupture of membranes (>18 hours before delivery) or suspected or proven maternal peri-partum infection.

Intra Uterine Growth Retardation/Growth restriction.

Respiratory: Respiratory Distress Syndrome (RDS), Meconium aspiration syndrome, Chronic Lung Disease (Oxygen-dependency beyond 28th day of life). Persistent pulmonary hypertension of the newborn.

Cardiovascular: Congenital Heart Disease (CHD), including treated Patent Ductus Arteriosus (PDA).

Gastrointestinal: Necrotizing Enterocolitis (NEC).

Surgical problems/Malformation including all the malformations and surgical problems with the exception of NEC and CHD, e.g. Gut and central nervous system (CNS) Malformations, Cleft Palate, Hydrocephalus (including post hemorrhagic hydrocephalus), Ambiguous Genitalia, etc.

Confirmed or suspected chromosomal/single gene/metabolic disorders.

Electrolyte/Glycaemia disorders including iatrogenic if requiring active management.

Neonatal immune deficiency, including haematological malignancies.

Neurological conditions including neonatal seizures, severe asphyxia, hypoxic-ischaemic encephalopathy etc.

Haematological disease including indirect hyperbilirubinaemia requiring treatment.

Toxicological problems, such as monitoring for neonatal abstinence syndrome.
